# Mapping the disparities in intimate partner violence prevalence and determinants across Sub-Saharan Africa

**DOI:** 10.3389/fpubh.2023.1188718

**Published:** 2023-06-28

**Authors:** Tilahun B. Mossie, Haile Mekonnen Fenta, Meseret Tadesse, Animut Tadele

**Affiliations:** ^1^Department of Psychiatry, School of Medicine, College of Medicine and Health Sciences, Bahir Dar University, Bahir Dar, Amhara Region, Ethiopia; ^2^Department of Statistics, College of Science, Bahir Dar University, Bahir Dar, Amhara Region, Ethiopia

**Keywords:** violence, intimate partner violence, disparities, domestic violence, Sub-Saharan Africa

## Abstract

**Objective:**

This study aimed to map disparities in prevalence and associated factors across countries in Sub-Saharan Africa.

**Methods:**

We used National Demographic and Health Survey (DHS) data from 26 countries in the region with 114,340 participants. Women and girls in the reproductive age group of 15 to 49 years were included in the study. To map disparities across countries and their provinces, we employed the kriging interpolation technique. We used STATA for data management.

**Result:**

The prevalence of physical, emotional and sexual IPV in Sub-Saharan Africa was 30.58, 30.22, and 12.6% respectively, and at least one form of IPV was 42.62%. Disparities were observed across the countries and provinces in each country. Younger age, secondary-level education and above, moderate participation in decision-making, not working out of home, not afraid of the spouse, rich (wealth index), not having a child, high maternal literacy, and rural residence relatively decreased the odds of IPV. The husbands’ lower education, alcohol consumption, and high controlling behavior increased the probability of IPV.

**Conclusion:**

The prevalence of Intimate Partner Violence in Sub-Saharan Africa is the highest in the world, a signal that the global agenda to end all forms of violence against women will be difficult to achieve. There is a large gap across countries and provinces in each country. Area-specific intervention packages that focus on modifiable factors should be strengthened.

## Introduction

Violence against women is ‘any act of gender-based violence that causes or is likely to cause physical, sexual or mental harm or suffering to women in public or private life, including threats of such acts, coercion or arbitrary deprivation of liberty’. It is a serious violation of human rights that poses a major obstacle to achieving gender equality that can occur in the family or within the community. It has a deep and long-term impact on women’s and girls’ lives, complicating their potential for growth, leadership, and prosperity (1).

Physical violence against women is one form of violence that involves hurting or trying to hurt a partner by hitting, kicking, burning, grabbing, pinching, shoving, slapping, hair-pulling, biting, denying medical care, or forcing alcohol and/or drug use, or using other physical force. It may also include property damage (2).

Sexual violence involves any sexual act committed against another person’s will, or when this person does not give consent or when consent cannot be given because the person is a child, has a mental disability, or is severely intoxicated or unconscious because of this alcohol or drug. It includes rape, sexual harassment, intimidation at work, trafficking and forced prostitution, corrective rape, and rape culture. Emotional violence comprises the erosion of a person’s self-esteem through constant criticism. Underestimating one’s abilities, attribution, or other verbal abuse; damage to relationships between partners and children; or not allowing a partner to see friends or family (2).

Intimate Partner Violence (IPV) comprises a behavior in an intimate relationship (married, single, and cohabiting) that causes physical, emotional, or sexual harm to the person in the relationship. It is any means of physical, emotional, or sexual assault/abuse or controlling behavior (3). IPV affects the physical and psychological health of women and their children. It is one of the global agendas set in the United Nations Sustainable Development Goals (UNSDG). The goal was set to eliminate all forms of violence against women and girls including trafficking and all forms of exploitation (4). Current reports by United Nations Women on the progress of SDG stated that the world is not on the way to achieving gender equality by 2030. According to this report more women and girls will remain in poverty in 2030 than today, which may contribute to an increment of violence against women, particularly in the third world (2–5).

Global studies reported that violence remains a significant problem with which the lifetime prevalence was 27% (23–31%) among women and girls in the reproductive age group (6, 7). Accordingly, lifetime experience of at least one form of violence is more common in Africa ranging from 27 to 44% than in other areas except for Oceania where the proportion was almost half (8). One year of experience (2018) of violence was 13% globally. Similarly, it was higher in Africa. African women and girls experienced physical, sexual, or both forms of violence at a proportion of 14 to 32% in one-year period in 2018 (8). The sub-Saharan region is a place where violence against women is higher. Almost one in five young women suffered from violence in about two-thirds of the countries (9, 10). The global and regional studies however did not include emotional violence in the study, and the stated prevalence could be higher than this.

Violence among women and girls is associated with different physical and psychological morbidity. Mental distress, depression, and anxiety are common among victims of violence. In addition, impairment of daily living was observed among the victims. A recent study in India reported that about 10% of women and girls who experienced violence had some functional difficulty at least in one of the functional domains (11–13).

Moreover, to end violence against women, evidence at the regional and national levels is important. It can help to plan specific activities based on the socio-demography and cultural background of the society that can be customized at least at the regional level of each nation. This study aimed to map the disparities in the prevalence of Intimate Partner Violence (IPV) among women and girls within and between Sub-Saharan Africa (SSA) countries based on the most recent Demographic and Health Survey Data. The regional and national proportion of IPV in the region was also analyzed.

## Methods

### Study design

Data for this study were obtained from the recent Demographic and Health Surveys (DHS)^1^ in 26 sub-Saharan African (SSA) countries. The choice of the 26 countries from SSA was based on the availability of the variables of interest for which the GPS coordinates (latitude and longitude) of household clusters were available. The DHS is the source of the country-level data from 26 countries with most recent surveys, and the studies were conducted between 2012 and 2021 (Table 1). We use DHS data because it is the largest source of data for low- and middle-income countries. A total of 114,340 women and girls in SSA were included in the study. More generally, the women’s files of the DHS were used. The DHS is a nationwide survey conducted across low middle income countries (LMICs) every 5 years. The DHS survey is representative of each LMIC and targets core maternal and child health indicators such as intimate partner violence. Multistage sampling was used to select the sample for each survey in various countries. Hence, the first step of the sampling procedure involved the selection of clusters (enumeration areas (EAs)), followed by systematic household sampling within the selected EAs. Variables: The outcome variable for this study includes single or multiple forms of physical, emotional, and sexual partner violence which was assessed using women’s self-reported responses to the questions depending on the modified Conflict of Tactic Scales of Status (14). Intimate Partner Violence (IPV) is defined as women who had experienced at least one event of physical, emotional, or sexual violence since the age of 15 years (14–16). The physical, sexual, and emotional violence had a Cronbach’s alpha of 0.81, 0.74, and 0.73 respectively, indicating overall good test performance of the interview questions. The different socio-demographic characteristics were taken as the independent variables for the different survey years, which were selected from different kinds of literature (17–23) (Table 2).

**Table 2:** The tools used to measure IPV in the demographic and health surveys, SSA, 2023.

Question/item	IPV type	Cronbach’s alpha
Push you, shake you, or throw something at you?	Physical IPV	0.81
Slap you?		
Twist your arm or pull your hair		
Punch you with his/her first or with something that could hurt you?		
Kick you, drag you, or beat you up?		
Try to choke you or burn you on purpose?		
Threaten or attack you with a knife, gun, or any other weapon?		
Physically force you to have sexual intercourse with him even when you did not want to?	Sexual IPV	0.74
Physically force you to perform any other sexual acts you did not want to?		
Force you with threats or in any other way to perform sexual acts you did not want to?		
Say or do something to humiliate you in front of others?	Emotional IPV	0.73
Threaten to hurt or harm you or someone close to you?		
Insult you or make you feel bad about yourself?		

Spatial heterogeneity of IPV prevalence: First we computed and mapped the crude prevalence of IPV in the 26 countries and 476 s-level administrative units (Provinces). The estimated prevalence of IPV in each country (and /or) state and we used ESRI Desktop 10.3 (24) to generate the maps of IPV prevalence using a kriging interpolation technique, a methodology widely used in spatial mapping (25–27).

### Statistical analysis

The data management was done using STATA (28). The data were weighted to make them representative and to provide better statistical estimates. We adopted the generalized linear mixed model (GLMM) (29–33) to examine the effect of the women, husband, and household characteristics on IVP measures for married women 15–49 age in SSA countries. The adopted GLMM model is:


$$g\left(\mu_{ijk}\right)=logit\left(\mu_{ijk}\right)=\log\left(\frac{\mu_{ijk}}{1-\mu_{ijk}}\right)=\log\left(\frac{P\left(y_{ijk}=1\right)}{P\left(y_{ijk}=0\right)}\right)=\eta_{ijk},$$

the $\mu_{ijk}$ and 1- $\mu_{ijk}$ are, respectively, the probability of women experiencing IPV and not experiencing IVP (i = 1,…,134, 340 women, j = 1,…,476 provinces, k = 1,…,26 countries) where $\beta_{0}$ is the log odds of intercept; $\beta_{1}$ … $\beta_{m}$ are effect sizes of women and household-level covariates (34–36).

The fully unconditional three-level model is written for the i^th^ women in the j^th^ provinces in the k^th^ country at the level I as


$$Level\;I:\eta_{ijk}=\pi_{ijk}+e_{ijk},$$

i=1,2,3,…,134,430,j=1,2,3,…,476andk=1,2,3,…,26.

for the j^th^ province in the kth country at level 2 as


$$Level\;II:\pi_{ijk}=\beta_{00k}+\gamma_{ijk,}$$

and for the kth country at level 3 as


$$\mathrm{Level}\;\mathrm{III}:\beta_{00k}=\gamma_{000}+\mu_{ook,}$$

where $e_{ijk}$ is a random effect women effect (level I residual), $\gamma_{0jk}$ is a random effect (level II residual), and $\mu_{00k}$ is a random effect (level III residual). The variance components among women within provinces, among provinces within countries, and countries are symbolized by $\sigma^{2}$, $\tau_{\pi }$, and $\tau_{\beta }$ respectively. Moreover, the intra-class correlation (ICC) for the three-level binary data is given for each of the levels separately.


$$\begin{array}{c} ICC_{provinces}\left(level\;2\right)=\frac{\sigma_{provinces}^{2}\left(\tau_{\pi }\right)}{\sigma_{countries}^{2}\left(\tau_{\beta }\right)+\sigma_{provinces}^{2}\left(\tau_{\pi }\right)+\frac{\pi^{2}}{3}}\\ \ldots ICC\;attributabletolevel\;2 \end{array}$$

$$\begin{array}{c} ICC_{countries}\left(level\;3\right)=\frac{\sigma_{countries}^{2}\left(\tau_{\beta }\right)}{\sigma_{countries}^{2}\left(\tau_{\beta }\right)+\sigma_{provinces}^{2}\left(\tau_{\pi }\right)+\frac{\pi^{2}}{3}}\\ \ldots ICC\;attributabletolevel\;3. \end{array}$$

## Results

**Figure 1:**
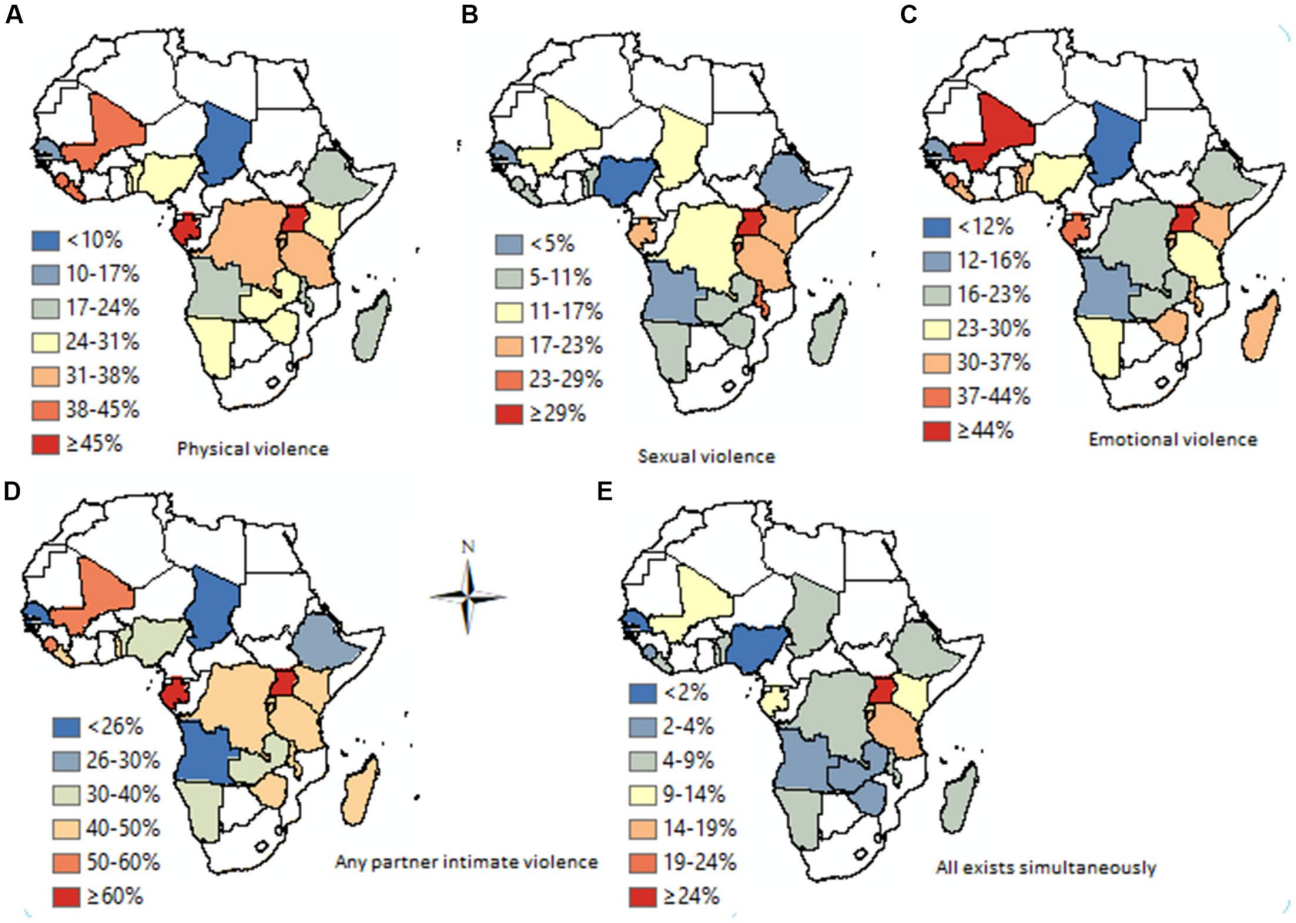
Prevalence of IPV across 26 countries.

The experience of different forms of intimate partner violence (IPV), single or multiple forms revealed that the 34,549 (30.22%), 34,963 (30.58%), and 14,402 (12.6%) of women reported that they experienced emotional, physical, and sexual IPV, respectively. However, 10,589 (9.26%) of the married women experienced both physical and sexual violence, and 8,373 (7.32%) women experienced all forms of intimate partner violence. Besides, the result revealed that about 48,723 (42.62%) of women experienced at least one form of intimate partner violence (Figure 1).

**Figure 2:**
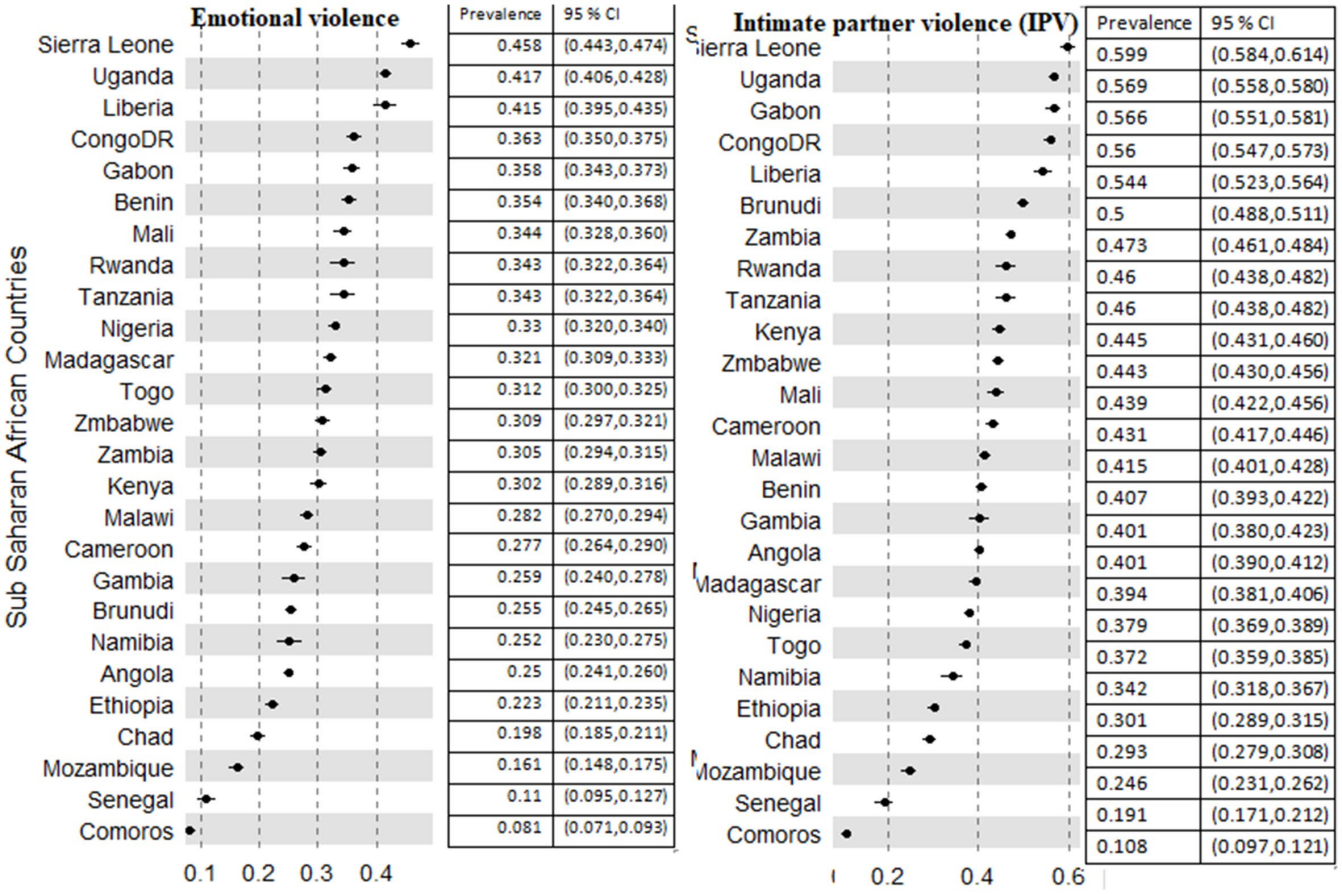
Prevalence of emotional and intimate partner violence among 26 countries in Sub-Saharan African countries.

There was a difference in the experience of IPV in Sub-Saharan Africa. The prevalence of IPV across countries ranges from 10.8% (95%CI; 9.7, 12.7%) to 59.9% (95% CI; 58.1, 61.4%). The least prevalence was recorded in Comoros (10.8%), yet it was the highest in Sierra Leone (59.9%) (Figure 2).

**Figure 3:**
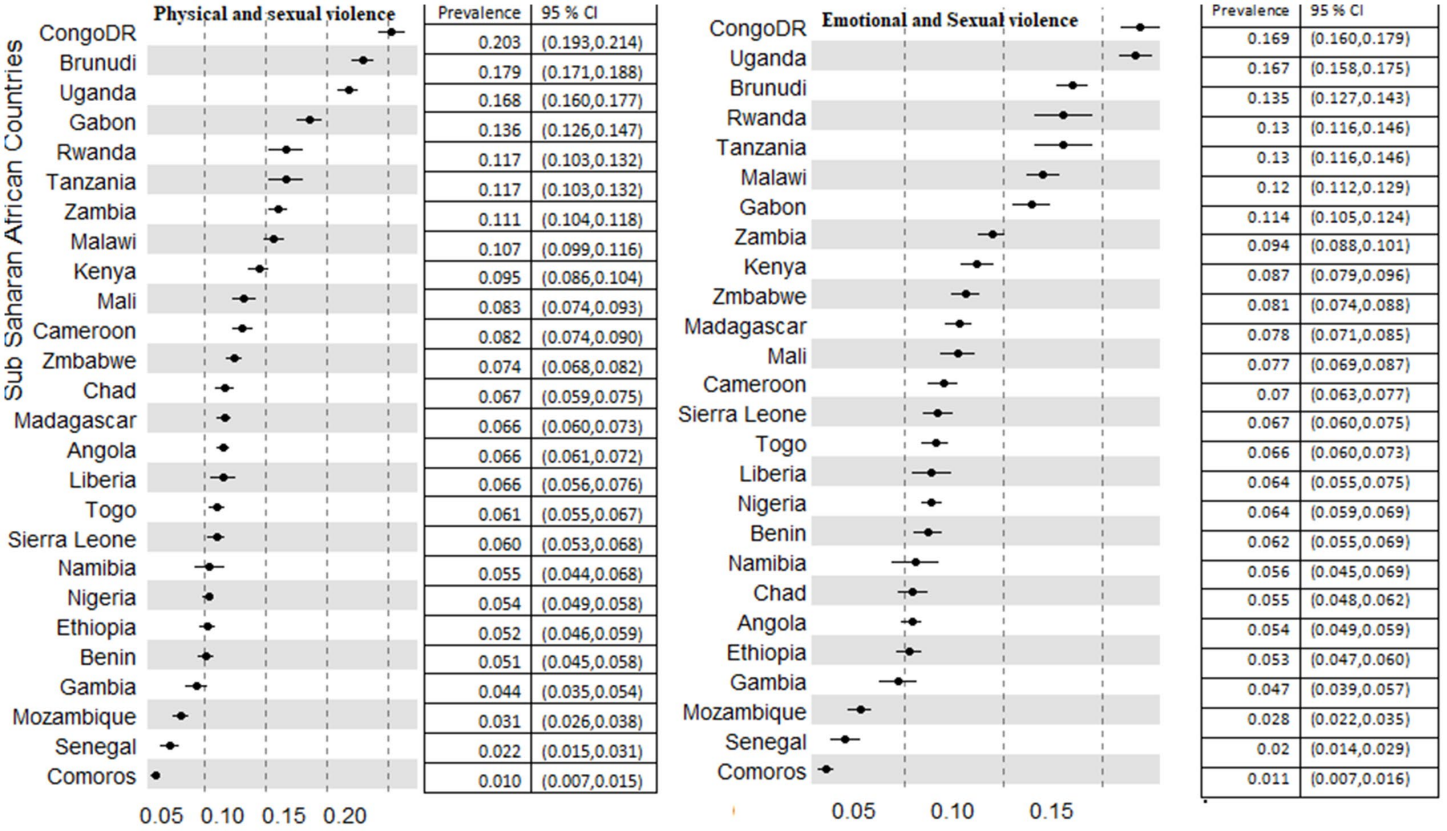
Prevalence of coexistence of different forms of violence among 26 countries in sub-Saharan African countries.

Experiencing two or more forms of IPV against women showed that the prevalence of both emotional and sexual forms of IPV was 8.77% in the region. The highest prevalence was 15, 16, and 17% in Burundi, Uganda, and the Democratic Republic of Congo (DR Congo) respectively. Comoros (1.6%), Senegal (2.5%), and Mozambique (3%) had relatively the least prevalence of both forms of IPV against women (Figure 3).

**Figure 4:**
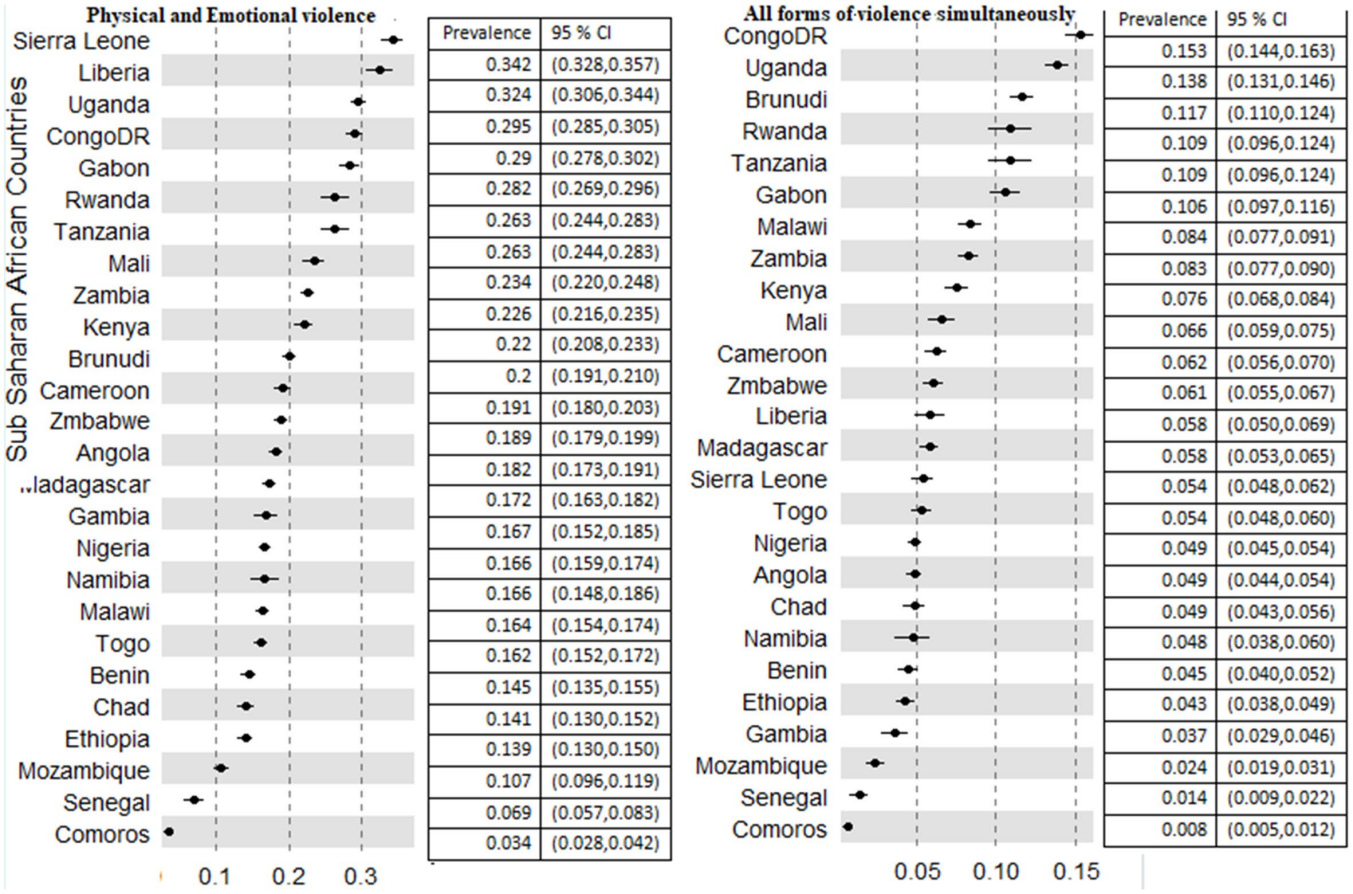
Prevalence of physical and emotional, and all forms of violence among 26 countries in sub-Saharan African countries.

DR Congo, Burundi, and Uganda had the highest prevalence of both forms of physical and sexual IPV in the region with a proportion of 20.3, 17.9, and 16.8%, respectively. The least prevalence was recorded in Comoros (1%), Senegal (2%), and Mozambique (3.1%). The prevalence of both physical and emotional IPV against women in Liberia (32.4%) and Sierra Leone (34.2%) was the highest in the region. Furthermore, in Senegal (6.9%) and Comoros (3.4%), the least prevalence of both physical and emotional IPV was recorded. All forms of IPV against women range from 0.8% in Comoros, 1.4%% in Senegal, and 2.4% in Mozambique to 11.7% in Burundi, 13.8% in Uganda, and 15.3% in DR Congo (Figure 4).

**Figure 5:**
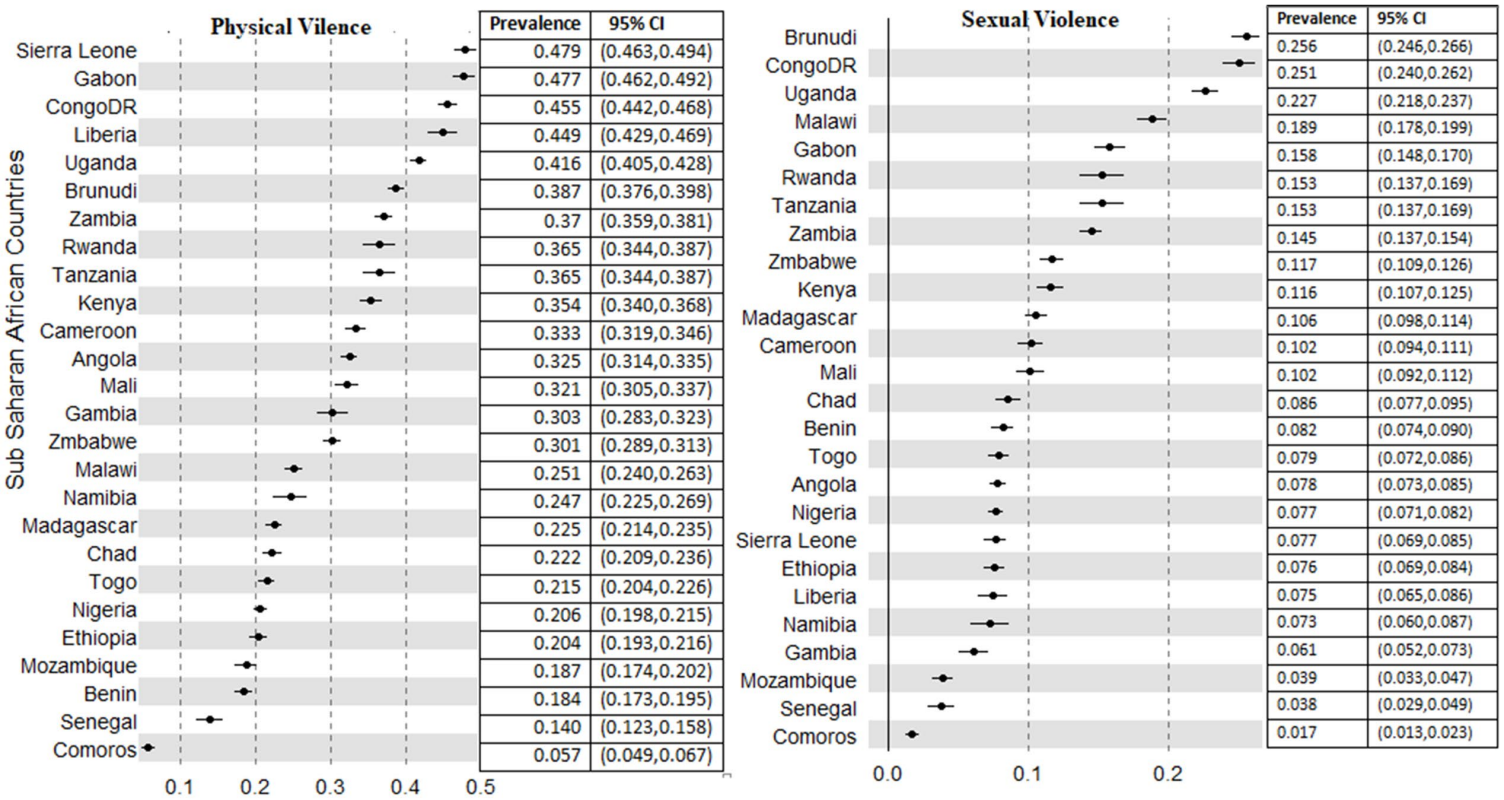
Prevalence of physical and sexual violence among 26 countries in sub-Saharan African countries.

The prevalence of different forms of IPV also revealed a discrepancy among the countries in Sub-Saharan Africa. The physical form of IPV against women ranges from 5.7% (95% CI; 4.9, 6.7%) in Comoros to 47.9% (95%CI; 46.3, 49.4%) in Sierra Leone (Figure 5). Across the provinces of the SSA nations, the physical form of IPV constituted 2 to 73%. The lowest prevalence was recorded in Jigawa province, Nigeria (2%), Matam, Senegal (4%), and Tibesti Chad (4%). The highest prevalence was 73% at Maracha, 70% at Katakwi (both in Uganda), and 70% at Kasai, Comoros (Table 1).

**Table 1:** Description of the sample size and the respective recent DHSs, Sub Saharan Africa, 2023.

Country	Survey year	Total women	Country	Survey year	Total women
Angola	2015	7,669	Malawi	2015	5,406
Benin	2018	4,488	Mali	2018	3,356
Burundi	2015	4,499	Mozambique	2015	2,871
Cameroon	2018	4,690	Namibia	2013	1,444
Chad	2014	3,776	Nigeria	2018	8,910
Comoros	2012	2,529	Rwanda	2019	1,947
Congo DR	2013	5,643	Senegal	2018	1,468
Ethiopia	2016	4,720	Sierra Leone	2019	4,055
Gabon	2012	4,147	Tanzania	2015	1,947
Gambia	2019	1,953	Togo	2013	5,343
Kenya	2014	4,488	Uganda	2015	7,536
Liberia	2019	2,331	Zambia	2018	7,358
Madagascar	2021	5,966	Zimbabwe	2015	5,800

The prevalence of sexual forms of intimate partner violence ranges from 1.7% (95% CI; 1.3, 2.3%) in Comoros to 25.6% (95% CI; 24.6, 26.6%) in Burundi (Figure 5). Among the administrative provinces in each nation, there was no finding of sexual violence among different provinces in Uganda (Butambala), Kenya (Wajir, and Garrisa), Chad (Tibesti), Nigeria (Kebbi), and Ethiopia (Somali) that was recorded as zero. Uganda (national prevalence of 22%) on the other hand had provinces with the highest form of sexual IPV, Budara (65%) and Ibanda (57%). In addition, Sankru province in Comoros recorded a 51% proportion of sexual IPV against women, one of the highest scores in the SSA.

The prevalence of an emotional form of IPV in the SSA region was in the range of 8.1% (95% CI; 7.1, 9.3%) in Comoros to 45.8% (95% CI; 44.3, 47.4%) in Sierra Leone (Figure 2). In addition, there is a wider difference across provinces. The lowest prevalence across the provinces was 3% (Lac, Chad), 4% at Tibesti, Ennedi (both in Chad), Garissa (Kenya), and Matam (Senegal). The provinces that scored the highest prevalence were Uganda [Buhweju (84%) and Ibanda (73%) and Nigeria (Kogi 73%)].

The description of IPV against women and girls by socio-demographic, household, and partner-level characteristics was computed. In the age category, the highest prevalence for IPV (43.68%), physical violence (31.44%), sexual violence (12.96%), and emotional violence (31.08%) was recorded in the age range of 25–34 years.

IPV by the level of education revealed that the highest prevalence was recorded among women who fall in the primary level of education. It was 46.92% for IPV, 37.10, 34.75, and 15.55% for physical, sexual, and emotional violence, respectively. As compared to Muslim and traditional religion followers, violence against women was the highest among Christians (45.56%). Besides, it was higher among women and girls who are not participating in decision-making (44.06%), women and girls who have exposure to media (40.89%), who are afraid of their husbands (73.40%), rural women (43.18%), women with the poorest wealth index (45.44%) and those who have four or more children (45.41%) (Table 1).

**Table 3:** The prevalence of partner violence by socio-demographic characteristics among ever-partnered or married 15–49-year-old women in SSA (*n* = 114,340).

Variables	Sample, *n* (%)	Physical, *n* (%)	Sexual, *n* (%)	Emotional, *n* (%)	IPV, *n* (%)
Outcome variables		34,963 (30.58)	14,402 (12.60)	34,549 (30.22)	48,723 (42.61)
Women level covariates					
Age in 5-year groups					
15–24	26,790 (23.43)	7,563 (28.23)	3,332 (12.44)	7,184 (26.82)	10,656 (39.78)
25–34	46,936 (41.05)	14,756 (31.44)	6,081 (12.96)	14,588 (31.08)	20,503 (43.68)
35–44	30,768 (26.91)	9,625 (31.28)	3,857 (12.54)	9,721 (31.59)	13,362 (43.43)
45–49	9,846 (8.61)	3,019 (30.66)	1,132 (11.50)	3,056 (31.04)	4,202 (42.68)
Education					
No education	38,858 (33.98)	10,813 (27.83)	4,212 (10.84)	11,057 (28.45)	15,301 (39.38)
Primary	42,425 (37.10)	14,742 (34.75)	6,599 (15.55)	13,861 (32.67)	19,907 (46.92)
Secondary	28,686 (25.09)	8,625 (30.07)	3,315 (11.56)	8,659 (30.19)	12,221 (42.60)
Higher	4,371 (3.82)	783 (17.91)	276 (6.31)	972 (22.24)	1,294 (29.60)
Religion					
Christian	62,155 (54.40)	21,228 (34.15)	8,211 (13.21)	19,849 (31.93)	28,315 (45.56)
Muslim	18,030 (15.78)	3,819 (21.18)	1,698 (9.42)	4,384 (24.32)	5,971 (33.12)
Traditional and others	34,069 (29.82)	9,903 (29.07)	4,489 (13.18)	10,296 (30.22)	14,415 (42.31)
Working status					
No	38,142 (33.40)	9,995 (26.20)	3,877 (10.16)	9,418 (24.69)	13,762 (36.08)
Yes	76,071 (66.60)	24,928 (32.77)	10,510 (13.82)	25,103 (33.00)	34,912 (45.89)
Women’s attitude to wife-beating					
Does not justify wife-beating	5,150 (4.50)	2,161 (41.96)	963 (18.70)	1,867 (36.25)	2,763 (53.65)
Justifies wife-beating	109,190 (95.50)	32,802 (30.04)	13,439 (12.31)	32,682 (29.93)	45,960 (42.09)
Women decision making					
No participation	32,640 (28.55)	10,806 (33.11)	4,835 (14.81)	11,046 (33.84)	14,675 (44.96)
Moderate participation	70,718 (61.85)	20,431 (28.89)	8,035 (11.36)	19,734 (27.91)	28,986 (40.99)
High level	10,982 (9.60)	3,726 (33.93)	1,532 (13.95)	3,769 (34.32)	5,062 (46.09)
Media exposure					
No	44,863 (39.30)	31,380 (30.05)	5,757 (12.83)	12,733 (28.38)	18,346 (40.89)
Yes	69,292 (60.70)	21,438 (30.94)	8,627 (12.45)	21,773 (31.42)	30,317 (43.75)
Women afraid husband					
Never afraid	57,132 (50.02)	10,276 (17.99)	3,854 (6.75)	10,327 (18.08)	16,355 (28.63)
Most of the time	14,005 (12.26)	8,697 (62.10)	4,386 (31.32)	8,250 (58.91)	10,280 (73.40)
Sometimes	43,081 (37.72)	15,950 (37.02)	6,145 (14.26)	15,935 (36.99)	22,039 (51.16)
Partner level covariates					
Husband’s education					
No education	30,691 (30.21)	8,184 (26.67)	3,215 (10.48)	8,494 (27.68)	11,741 (38.26)
Primary	30,715 (30.23)	10,236 (33.33)	4,648 (15.13)	9,722 (31.65)	14,172 (46.14)
Secondary	32,199 (31.69)	10,197 (31.67)	3,875 (12.03)	9,758 (30.31)	14,090 (43.76)
Higher	7,989 (7.86)	1,636 (20.48)	575 (7.20)	1,839 (23.02)	2,585 (32.36)
Partner’s controlling behavior					
No controlling behavior	42,840 (37.47)	6,151 (14.36)	2,073 (4.84)	5,104 (11.91)	9,212 (21.50)
Has controlling behavior	71,500 (62.53)	28,812 (40.30)	12,329 (17.24)	29,445 (41.18)	39,511 (55.26)
Husband alcohol use					
No	70,273 (61.47)	14,977 (21.31)	5,931 (8.44)	15,817 (22.51)	22,791 (32.43)
Yes	44,039 (38.53)	19,981 (45.37)	8,467 (19.23)	18,726 (42.52)	25,926 (58.87)
Household level covariates					
Place of residence					
Urban	37,475 (32.78)	11,231 (29.97)	4,114 (10.98)	11,133 (29.71)	15,530 (41.44)
Rural	76,865 (67.22)	23,732 (30.87)	10,288 (13.38)	23,416 (30.46)	33,193 (43.18)
Wealth Index					
Poorest	16,706 (23.36)	9,009 (33.73)	3,697 (13.84)	8,625 (32.30)	12,135 (45.44)
Poorer	23,829 (20.84)	7,770 (32.61)	3,282 (13.77)	7,458 (31.30)	10,616 (44.55)
Middle	22,553 (19.72)	6,964 (30.88)	2,884 (12.79)	6,911 (30.64)	9,763 (43.29)
Richer	21,345 (18.67)	6,302 (29.52)	2,631 (12.33)	6,338 (29.69)	8,949 (41.93)
Richest	19,907 (17.41)	4,918 (24.70)	1,908 (9.58)	5,217 (26.21)	7,260 (36.47)
Total number of children					
0	6,584 (5.76)	1,287 (19.55)	613 (9.31)	1,382 (20.99)	2,018 (30.65)
1–4	55,202 (48.28)	16,204 (29.35)	6,639 (12.03)	16,024 (29.03)	22,838 (41.37)
4 or more	52,554 (45.96)	17,472 (33.25)	7,150 (13.61)	17,143 (32.62)	23,867 (45.41)

### Factors associated with experiencing intimate partner violence

We computed a multiple logistic regression to identify factors associated with Intimate Partner Violence against women and girls in Sub-Sahara Africa. Age, education, participation in decision-making, current working status, afraid of partners, wealth index, residence, and having a child were significantly associated with violence against women. Among spouse factors, the partner’s level of education, controlling behavior, and alcohol use showed significant association. Among community-level factors, maternal illiteracy had a significant association with IPV.

Women and girls in SSA whose age ranges from 25–34 years had relatively higher odds of IPV [AOR = 1.07 (1.02, 1.11)] than younger women (of 15–24 years). However, a higher education beyond the secondary level [AOR = 0.78 (0.70, 0.86)] as compared to no formal education, and moderate participation in decision-making [AOR = 0.95 (0.91, 0.99)] than no participation, relatively decreased the odds of IPV by 22 and 5%, respectively.

Higher odds of IPV were seen among women who were working [AOR = 1.15 (1.11, 1.19)] than not working; who reported high participation in decision making [AOR = 1.15 (1.11, 1.19)] than women who did not participate; afraid of their husbands most of the time [AOR = 5.06 (4.80, 5.33)] than who did not afraid; sometimes afraid of their husbands [AOR = 2.46 (2.38, 2.54)] than who did not afraid.

Among husband-related factors, higher probabilities of IPV were found among women whose husbands had a primary level of education [AOR = 1.06 (1.02, 1.11)] than their counterparts with no formal education; whose partner had a higher controlling behavior [AOR = 3.77 (3.64, 3.90)] than not controlling; and whose husband drank alcohol [AOR = 2.44 (2.36, 2.52)] than did not drink alcohol. However, when husbands’ education level was higher than the secondary level, the probability of IPV decreased by 21% [AOR = 0.79 (0.73, 0.85)] than their counterparts with no education.

A higher wealth index was inversely associated with IPV. Richer and Richest women had relatively lower odds of IPV [AOR = 0.94 (0.89, 099)], [AOR = 0.81 (0.75, 0.86)] than the poorest women. As compared to women who did not have any child, there were higher odds of IPV among women with one up to three children [AOR = 1.50 (1.40, 1.61)] and women with four or more children [AOR = 1.80 (1.67, 1.94)].

In Sub-Sahara Africa where there was a high proportion of maternal illiteracy, the odds of IPV were higher [AOR = 1.33 (1.14, 1.55)] than in areas with a low proportion of maternal illiteracy. Rural women also had a relatively lower probability of IPV [AOR = 0.95 (0.91, 0.99)] than urban women. Yet there was no statistically significant difference among the geographical regions of SSA.

### Random effect estimates

Table 4 describes the result of the multilevel regression for empty (Model 0), community (Model I), individual model (model III), and individual and community (Model 4) level factors to measure the random effect of community and fixed effect of covariates associated with intimate partner violence (IPV) among women aged 15–49 years in sub-Saharan African countries. The IPV in 134,430 women from 476 provinces in 26 countries of SSA regions was included in the study. The final model, as expected, has a much better model fit than the other models (much less AIC = 110,261), since it incorporates women, province, and country-level covariates. The estimated variance of random effects due to country level and province level were 0.02 and 0.05, respectively. Moreover, the estimated intra-class correlation for country and province level is: 4 and 10%, respectively, implying that 4 and 10% of the total variation of IPV among 15–49 years old women was explained by countries and provinces, respectively.

**Table 4:** Factors associated with experiencing physical, emotional, sexual, and intimate partner violence (IPV), SSA, 2023.

Women level covariates	Model 0 AOR (95% CI)	Model I AOR (95% CI)	Model II AOR (95% CI)	Model III AOR (95% CI)
Age in 5-year groups				
15–24	1	1	1	
25–34			1.07 (1.02, 1.11)*	1.07 (1.02, 1.11)**
35–44			1.01 (0.94, 1.05)	0.99 (0.95, 1.05)
45–49			1.01 (0.94, 1.08)	1.02 (0.94, 1.07)
Education				
No Education			1	
Primary			1.03 (0.99, 1.08)	1.03 (0.98, 107)
Secondary			0.98 (0.93, 1.03)	0.97 (0.92, 1.02)
Higher			0.78 (0.71, 0.87)***	0.78 (0.70, 0.86)***
Religion				
Christian	1		1	
Muslim			0.92 (0.86, 0.97)***	0.93 (0.87, 0.98)
Traditional And Others			0.97 (0.92, 1.02)	0.97 (0.93, 1.03)
Respondent Currently Working				
No	1	1	1	1
Yes			1.15 (1.11, 1.22)***	1.15 (1.11, 1.19)***
Women decision making				
No participation	1		1	
Moderate participation			0.95 (0.92, 0.99)*	0.95 (0.91, 0.99)**
High level			1.15 (1.08, 1.21)***	
Media exposure				
No	1		1	
Yes			1.16 (1.12, 1.20)***	1.15 (0.11, 1.20)***
Women afraid husband				
Never afraid	1	1	1	
Most of the time			5.05 (4.80, 5.33)***	5.06 (4.80, 5.33)***
Sometimes			2.45 (2.37, 2.54)***	2.46 (2.38, 2.54)***
Partner level covariates				
Husband’s education				
No education	1	1	1	1
Primary			1.07 (1.02, 1.12)***	1.06 (1.02, 1.11)*
Secondary			0.98 (0.94, 1.03)	0.98 (0.93, 1.03)***
Higher			0.79 (0.74, 0.86)***	0.79 (0.73, 0.85)
Partner’s controlling behavior (MC)				
No controlling behavior	1	1	1	
Has controlling behavior			3.77 (3.64, 3.90)***	3.77 (3.64, 3.90)***
Husband alcohol use				
No	1	1		
Yes			2.45 (2.37, 2.53)***	2.44 (2.36, 2.52)***
Household level covariates				
Wealth Index				
Poorest	1	1	1	
Poorer			0.99 (0.95. 1.04)	0.99 (0.94, 1.03)
Middle			0.97 (0.92. 1.02)	0.96 (0.91, 1.10)
Richer			0.96 (0.91, 1.01)	0.94 (0.89, 099)**
Richest			0.84 (0.79, 0.89)***	0.81 (0.75, 0.86)***
Total number of children				
0	1		1	1
1–3			1.50 (1.41, 1.61)***	1.50 (1.40, 1.61)
4 and more			1.80 (1.67, 1.93)***	1.80 (1.67, 1.94)***
Community level factors				
Community level poverty				
Low		1		1
High		0.82 (0.73, 0.92)**		0.94 (0.84, 1.05)
The proportion of maternal illiteracy				
Low		1		
High		1.40 (1.19, 1.64)***		1.33 (1.14, 1.55)***
The proportion of media exposure				
Low		1		
High		1.27 (1.08, 1.49)**		1.12 (0.96, 1.31)
Place of residence				
Urban		1		1
Rural		1.05 (1.02, 1.08)**		0.95 (0.91, 0.99)*
Regions				
Central Africa		1	1	1
East Africa		0.78 (0.45, 1.35)		1.02 (0.66, 1.58)
South Africa		0.53 (0.17, 1.64)		0.78 (0.3, 1.91)
West Africa		0.93 (0.51, 1.71)		1.24 (0.77, 2.01)
Random effect components				
Country				
Variance (95% CI)	0.08 (0.02, 0.29)		0.02 (0.01, 0.02)	0.02 (0.01, 0.09)
ICC (95% CI)	0.07 (0.04, 0.13)	0.07 (0.04, 0.12)	0.04 (0.02, 0.08)	0.04 (0.02, 0.08)
*N*	25	25	25	25
Provinces				
Variance (95% CI)	0.07 (0.05,0.10)		0.05 (0.04, 0.07)	0.05 (0.03, 0.06)
ICC (95% CI)	0.14 (0.11, 0.19)	0.13 (0.10, 0.18)	0.10 (0.08, 0.14)	0.10 (0.08, 0.13)
N	476	**476**	476	476
Model fit				
LR test	9,188.5***	8,608.3***	4,959.2***	4,901.7
Wald chi-square	Reference	44.23***	15,431.5***	15,449.5****
-Log-likelihood	74,183.2	74,161.6	55,108.3	55,095.4***
AIC	148,372.3	148,343.3	110,272.5	110,260.7

*p < 0.05, **p < 0.01, ***p < 0.001. N, Sample size; 1 = Reference category; ICC, Intra-Class Correlation; LR Test, Likelihood ratio Test; AIC, Akaike’s Information Criterion.

## Discussion

In this study, we determined the prevalence of the different forms of IPV against women and girls in the reproductive age group, 15 to 49 years old in 26 countries in Sub-Saharan Africa. We used the latest demographic data. The proportion of women and girls that experienced emotional, physical, and sexual intimate partner violence was 30.22, 30.58, and 12.6%, respectively. According to prior studies, this is the highest figure across the globe except in Oceania (8, 37). We noted that the regional prevalence lies from about 10.8% in Comoros to about 59.9% in Sierra Leone. And it is almost similar to prior studies conducted a decade ago (38).

In the current study, we tried to further determine the prevalence for the provinces of the countries based on the demographic survey. This might help stakeholders to plan specific interventions in the provinces in a certain country since there is a diverse experience among provinces in each country. The physical form of IPV ranged from 5.7% (Comoros) to 47.9% (Sierra Leone). Based on the province-level demographic data analysis, the prevalence ranges from 2% (Jigawa, Nigeria) to 73% (Maracha, Uganda). Although the national prevalence of physical IPV in Comoros was low, it has a province called Kasai that recorded the highest prevalence (70%) of physical IPV. The gaps in the prevalence can be a result of different cultural perspectives across the provinces. There may be cultural facilitators and barriers to IPV. Hence, at least sharing experiences across provinces in each country can be helpful to narrow the disparities. Adapting culturally acceptable approaches may be the secondary benefit of such experiences. Culturally appropriate interventions were effective to address IPV in rural areas (39).

The prevalence of the emotional type of IPV ranged from 8.1% in Comoros to 45.8% in Sierra Leone. The sub-national analysis showed that across the provinces in SSA, there was a huge gap in the prevalence. It was in the range of 3% (Lac, Chad) to 73% (Kogi, Nigeria). The ecological framework of violence views it as an ‘interplay among personal, situational and socio-cultural contexts.’ The huge variation across countries and their provinces in SSA can stem from those factors. Intervention packages aimed at violence against women and girls shall incorporate components dealing with those important factors (40).

The sexual form of IPV was also common in the SSA region. The least prevalence was 1.7% in Comoros whereas Burundi had the highest prevalence (25.8%). Across the provinces, some areas did not report any sexual violence. Those provinces were from Uganda (Butambala), Kenya (Wajir, and Garrisa), Chad (Tibesti), Nigeria (Kebbi), and Ethiopia (Somali). The highest prevalence was in Uganda [Budara (65%) and Ibanda (57%)]. Like the above forms of IPV, the difference in the prevalence of sexual IPV across nations and provinces is very high. To lessen the burden of IPV, analyzing the role of gender power, evidence-based practices, sustainable investment through an inter-sectoral approach, and promoting aspirational thoughts of activism are also helpful (41). Peer norm targeted interventions, early prevention of sexual violence and consensus interventions for the youth girls may be effective in addressing sexual IPV among girls (42).

The prevalence of two or more forms of IPV against women was high in the SSA. Emotional and sexual IPV co-existed together among 8.77% of women and girls with a greater variation among countries from 1.6% in Comoros to 17% in DR Congo. Physical and sexual forms of IPV also co-occurred together among 9.26% of women and girls in the region. Emotional and physical forms of IPV were the highest prevalent form of co-occurring IPV, with a 20.07% rate of proportion. Among SSA countries, the prevalence lies from 3.5% in Comoros to 36% in Sierra Leone. The prevalence of all the 3 forms of IPV against women and girls was also very high. About 7.32% of participants experienced all forms of violence in the region. There is a variation across countries, ranging from 1% in Comoros to 16% in DR Congo.

Violence is challenging the health costs of low-income countries, mainly SSA, and hindering development (43, 44). A dedicated room for IPV in the community, using role models among the leaders of violence against women, and community perceptions of violence as an issue worth are important to combat all forms of IPV in low- and middle-income countries (45). Moreover, intervention packages against IPV in SSA shall focus on the modifiable risk factors to prevent it, which include unplanned pregnancy, literacy, unmarried women and girls, and younger girls (46).

Age, education, co-determination, current work status, fear of a partner, wealth index, place of residence, and desire to have children were significantly associated with IPV against women. Among the spousal factors, the partner’s education level, behavioral inhibition, and alcohol consumption showed significant associations. Among the community-level factors, maternal illiteracy was significantly associated with experiencing IPV.

The odds of IPV were less likely among younger aged 15 to 24 years than 25 to 34 years old women. It could be because older girls, especially those above 21 years old, were more likely to have multiple psycho-social problems and different forms of violence than younger ones (47–49).

Higher education of secondary level and above than no education, and moderate participation in decision making than no participation relatively decreased the proportion of IPV. Protective behavioral strategies can be used by women and girls when they are educated and have a chance to discuss with their partners (50). Also, joint decision making can contribute to the decrement in the proportion of IPV by empowering women and girls (48, 49). The controlling behavior of husbands was associated with a higher probability of IPV in the current study. This may be related to restricting spouses’ activities at work and decision-making (51). This behavior has direct implications for urban women who had a higher probability of IPV in SSA.

The husband’s literacy, particularly the secondary level of education and above was protective against IPV as compared to no education. Partner behavior of drinking alcohol was associated with higher odds of IPV. Drinking can influence behavior. And such husbands had a higher probability of performing IPV (52). This suggests the involvement of spouses in intervention and programs that focus on IPV (53).

The richest women in terms of wealth and women who had no child had lower odds of IPV in this study. IPV can cross boundaries in the region. Similar prior studies across 20 and 40 countries, respectively, revealed that except for Mozambique in the first study, wealthier women had lower odds of IPV in both studies (23, 54) and the number of children is a modifiable factor that needs to be considered by programs against IPV.

Moreover, IPV remains a day-to-day challenge to women and girls in Sub-Saharan Africa as it has the highest prevalence in the world (excluding Oceania). This is a signal that the region is not going well toward the global SDG goal to eliminate all forms of violence against women and girls. The study seeks to map the prevalence across countries and their states. There are large differences between countries and provinces in the experiences of IPV. Policymakers and other stakeholders should, therefore, consider region-specific interventions against IPV. In addition, several sociodemographic, spousal, and community-based modifiable factors need to be considered to plan effective and sustainable interventions.

### Implications for future research

Contextualized, adapted and more complex interventions to prevent IPV in SSA shall be developed for a trial. Such interventions that involve women with lived experience, husbands, experts (including researchers), community leaders, stakeholders and policy makers can be feasible interventions. Hence, researchers need to focus on developing such trials.

### Implications for policy and practice

The intervention packages planned to prevent IPV against women and girls shall be contextualized to the local context in each province across countries.

### Strength and limitation of the study

The study included 114,340 participants from 26 countries. And it reveals the three types of IPV against women and girls. We consider these as the strengths of the study. On the other hand, the study period across countries varies from 2012 to 2021. In countries that did not update their DHS data until 2015 (7 countries), the current findings may not reflect the real situation. This is the limitation of the study.

## Data availability statement

The original contributions presented in the study are included in the article/Supplementary material, further inquiries can be directed to the corresponding author.

## Ethics statement

Ethical review and approval was not required for the study on human participants in accordance with the local legislation and institutional requirements. The patients/participants provided their written informed consent to participate in this study.

## Author contributions

TM designed the draft protocol, drafted the design and methodology, accessed the raw data, verified data reported in the manuscript, interpreted the findings, and writeup the manuscript. HF designed the draft protocol, drafted the design and methodology, analyzed the data, accessed the raw data and verified data reported in the manuscript, interpreted the finding, and write up the manuscript. MT drafted the design and methodology, interpreted the findings, and writeup the manuscript. AT designed the draft protocol, drafted the design and methodology, writeup the manuscript, and edit the manuscript. All authors confirm that we had access to the data in the study and accept responsibility for publication.

## Conflict of interest

The authors declare that the research was conducted in the absence of any commercial or financial relationships that could be construed as a potential conflict of interest.

## Publisher’s note

All claims expressed in this article are solely those of the authors and do not necessarily represent those of their affiliated organizations, or those of the publisher, the editors and the reviewers. Any product that may be evaluated in this article, or claim that may be made by its manufacturer, is not guaranteed or endorsed by the publisher.

## Footnotes

^1^ https://dhsprogram.com
