# Supplementary material for: Mapping the disparities in intimate partner violence prevalence and determinants across Sub-Saharan Africa
Source: Front Public Health. 2023 Jun 28;11:1188718. doi: 10.3389/fpubh.2023.1188718 (PMC10337829; doi:10.3389/fpubh.2023.1188718)
Supplement: Supplementary file 1 [file Data_Sheet_1.PDF]

Country	Regions	total sample	Prevalence of Physical IPV	Prevalence of Sexual IPV	Prevalence of Emotional IPV	Prevalence of IPV
Uganda	BUHWEJU	25	68	32	84	88
	KATAKWI	37	70	30	49	86
	KIBUKU	54	59	50	65	83
	BUDAKA	34	56	65	56	82
	IBANDA	49	61	57	73	82
	MARACHA	59	73	20	63	80
	KIRUHURA	72	43	26	68	79
	LAMWO	40	65	15	58	78
	BULAMBUL	22	55	27	64	77
	KAMWENG	57	56	30	67	77
	LYANTOND	13	54	31	62	77
Comoros	Kasai	259	70	41	55	77
	BUTALEJA	64	50	50	58	77
	Sankuru	180	66	51	71	76
Nigeria	GOMBE	259	51	45	71	76
	KOGI	183	27	8	73	75
	ISINGIRO	84	46	23	70	75
Sierra Leon	North West	658	64	13	59	74
	MOROTO	50	52	20	58	74
	BUSHENYI	50	48	48	52	74
	KYEGEGWA	69	48	41	58	74
Brunudi	Kirundo	352	61	39	38	73
Kenya	Maryland	156	60	7	53	72
	NGORA	43	60	23	56	72
	SHEEMA	50	44	14	66	72
Kenya	Nyamira	99	48	22	66	72
	ABIM	31	55	35	52	71
	AMOLATAR	20	50	40	65	70
	BUKEDEA	70	56	34	43	70
	TORORO	142	54	45	50	70
	MOYO	36	53	19	61	69
	SERERE	62	56	31	53	69
	KAABONG	62	55	10	50	69
	KITGUM	62	53	11	44	69
	BUNDIBUG	26	46	35	54	69
	BUKOMANS	13	46	23	31	69
	Kasai-Centr	227	59	34	41	69
	Sinoe	134	60	10	53	69
	Tshopo	183	58	32	40	68
	NAKAPIRIPI	66	44	39	38	68
	AMUDAT	31	65	26	39	68
	BUSIA	96	47	32	49	68
	MBARARA	99	43	23	62	68
	NTUNGAM	105	45	37	55	68
	RUKUNGIRI	104	45	24	53	67
	KOLE	70	57	17	36	67
	Muramvya	221	46	44	33	67

	KALIRO	24	33	38	33	67
Zambia	Muchinga	695	52	26	43	66
Gabon	Ogooue-Ivin	465	59	20	41	66
	Cankuzo	225	49	32	36	66
	Western	526	56	10	55	66
	APAC	79	58	22	52	66
	PALLISA	86	47	43	44	65
	PLATEAU	212	24	16	59	65
	Bungoma	108	53	39	44	65
	Ngounie	423	53	21	49	65
	Ogooue-Lol	349	53	16	36	64
	Rumonge	217	42	41	41	64
	Migori	123	52	24	49	64
	BULIISA	25	48	20	40	64
	Lomami	233	55	29	42	64
	MITOOMA	36	42	22	58	64
	ARUA	163	45	22	51	64
	KABALE	179	45	31	51	64
	BAUCHI	291	22	23	57	64
	OYAM	101	61	18	47	63
Angola	Malanje	423	56	17	43	63
	RUBIRIZI	24	29	33	58	63
	Vihiga	82	46	10	54	62
	KYANKWAN	37	49	22	38	62
	EDO	145	37	11	59	62
	YUMBE	68	41	26	51	62
	Equateur	175	49	19	38	61
	KUMI	59	41	20	47	61
	LIRA	110	47	29	46	61
	Kisumu	92	54	14	45	61
	Estuaire	383	51	20	39	61
	Narok	120	55	18	23	61
	Nimba	217	47	18	55	61
Cameroon	CENTRE	426	55	21	39	61
	SOROTI	89	43	16	35	61
	Nord-Ubang	187	49	21	49	60
	Kwilu	299	50	29	39	60
	Grand Cape	145	52	11	45	60
	AMURIA	70	46	14	40	60
	MANAFWA	77	56	25	32	60
	Sud-Ubangi	181	54	23	34	60
	KABAROLE	109	40	29	47	60
	Northern	1020	48	9	43	59
	ZOMBO	54	44	20	52	59
	KYENJOJO	88	34	27	49	59
Tanzania	Kigoma	85	52	24	42	59
	Kasai-Orient	238	53	26	34	59
	Busia	99	44	15	49	59
	MAYUGE	222	40	29	41	59

Madagascar	ANTANANA	398	42	22	49	59
	Samburu	106	52	8	41	58
	KOBOKO	24	42	25	46	58
	KAYUNGA	36	44	11	42	58
	Lunda Norte	422	51	16	33	58
	VAKINANKA	265	37	22	46	58
Mali	BAMAKO	419	39	17	45	58
	SSEMBABUI	38	50	29	50	58
	MPIGI	38	53	26	47	58
	EBONYI	285	44	17	46	58
	SEGOU	451	49	14	43	58
Chad	Tandjile	185	50	16	43	58
	KADUNA	313	20	10	57	58
	River Gee	122	47	1	40	57
	Kayanza	242	45	27	26	57
	Kericho	89	42	8	53	57
	AGAGO	89	55	6	30	57
	ADAMAWA	220	34	15	56	57
	Luapula	774	44	20	34	57
	GULU	133	51	8	41	57
	Nyanga	369	46	20	40	57
	ADJUMANI	46	41	17	46	57
	Haut-Lomar	163	45	29	34	56
	AMURU	48	48	6	35	56
	NIGER	287	34	14	43	56
	Nairobi	123	43	21	36	56
	Tshuapa	175	46	33	37	56
	Tanganyka	170	47	24	43	56
	RAKAI	95	39	26	42	56
	NAMAYING	70	50	19	39	56
	Montserrado	264	45	5	39	56
	Mai-Ndomb	218	46	29	30	56
	Mara	83	41	14	46	55
	MITYANA	56	46	21	41	55
	Kirinyaga	87	47	7	40	55
	ANALAMAN	243	32	16	47	55
	Woleu-Nten	354	44	18	41	55
	Eastern	858	41	5	40	55
	NWOYA	40	33	25	48	55
	Makueni	102	47	8	39	55
	JINJA	73	34	29	48	55
	ANAMBRA	307	31	6	47	55
	LUUKA	22	36	18	50	55
	NAMUTUM	22	41	36	45	55
	Haut-Katang	176	45	15	33	55
	Homa Bay	114	44	14	39	54
	SIRONKO	81	41	21	32	54
	Bong	186	43	9	46	54
	Gbarpolu	129	42	3	46	54

	Copperbelt	790	45	19	34	54
	Kinshasa	327	45	14	31	54
	Grand Bassa	170	46	8	45	54
	MUBENDE	85	35	21	39	54
	Embu	85	40	12	34	54
	KAMULI	63	30	19	32	54
	ALEBTONG	52	54	19	29	54
Zmbabwe	Mashonalan	664	39	17	34	53
	KOULIKORO	477	44	10	41	53
	NEBBI	94	41	21	30	53
	Namibe	373	40	13	36	53
	KALUNGU	17	41	18	41	53
	Benguela	461	37	9	38	53
	Haut-Uele	155	41	16	39	53
	Moyen-Ogo	329	47	14	31	53
	RIVERS	238	40	12	38	53
	Mongala	185	42	31	35	52
	HAUTE MA	231	29	13	42	52
	ITASY	284	37	18	39	52
	Southern	993	39	4	40	52
	EST	436	42	11	33	52
Benin	Plateau	292	12	10	48	52
Gambia	Janjanbureh	245	42	11	36	52
	Kilimanjaro	83	41	19	37	52
Namibia	Kavango	149	39	12	34	52
	IGANGA	60	33	28	37	52
	Maniema	298	39	26	29	51
	MASINDI	76	39	17	33	51
	Trans-Nzoia	78	37	13	44	51
	OUEST	433	31	9	39	51
	KANUNGU	82	46	21	41	51
	PADER	43	47	7	28	51
	NULL	707	42	14	31	51
	Tana River	102	45	12	31	51
Togo	Savanes	1053	31	10	47	51
	SUD	357	43	12	35	51
	Tanga	73	40	11	37	51
	TARABA	229	24	9	42	51
	Kisii	115	43	19	25	50
	MBALE	125	45	21	33	50
	Ngozi	375	43	18	28	50
	BENUE	293	32	2	45	50
	Ruvuma	78	40	18	44	50
	KIBOGA	28	39	21	39	50
	NAKASEKE	22	27	14	27	50
	BUKWO	8	38	25	25	50
	Haut-Ogoou	411	43	9	26	50
	Mayo Kebbi	197	42	9	37	50
	Logone Orie	189	40	5	32	50

	Grand Kru	119	47	4	19	50
	Kitui	99	44	15	33	49
	Dodoma	97	40	16	39	49
	Nakuru	97	34	12	38	49
	Eastern	947	36	13	34	49
	Bomi	120	41	8	37	49
	Bulawayo	401	29	9	35	49
	Kakamega	110	41	13	35	49
	Lualaba	147	37	21	24	49
	DELTA	172	26	7	48	49
	Shinyanga	86	41	17	38	49
	KABERAMA	43	42	12	28	49
	Gitega	300	39	25	24	49
	Njombe	80	41	20	34	49
	Karusi	293	42	15	16	49
	Sud-Kivu	300	35	19	37	49
	Meru	111	39	10	38	49
	Tharaka-Nit	72	31	15	33	49
	Nord-Kivu	301	24	26	33	49
	Southern	734	38	10	33	49
	Mashonalan	565	37	11	28	48
	BONGOLAV	286	29	17	37	48
Rwanda	West	456	38	15	32	48
	KOTIDO	54	30	13	39	48
	Mayo Kebbi	181	36	18	31	48
	Huila	489	38	8	35	48
	Northern	743	36	18	32	48
	Manyara	90	38	12	36	48
	Atacora	344	24	7	42	48
	NTOROKO	21	19	10	33	48
	NAPAK	42	33	10	29	48
	KAYES	468	41	15	37	47
	Rukwa	78	35	17	36	47
	South	477	39	16	36	47
	KIRYANDOI	57	46	9	21	47
	Katavi	91	36	15	37	47
	Harare	699	32	12	33	47
	BUDUDA	49	41	27	27	47
	Midlands	616	31	11	34	47
	KAPCHORV	30	27	17	40	47
	BUGIRI	43	42	28	35	47
	Bururi	153	22	30	22	46
	Cibitoke	274	33	30	22	46
	AMORON I	264	27	15	40	46
	Mbeya	76	32	14	30	46
	Laikipia	87	36	14	30	46
	Ogooue Ma	357	41	6	27	46
	Kwango	242	39	30	24	46
	DOKOLO	48	35	13	38	46

Mtwara	72	36	19	32	46
North	317	39	15	32	46
Manicaland	661	32	15	31	46
NORD-OUE	270	24	13	38	46
Bubanza	261	37	24	30	45
BUVUMA	88	33	14	27	45
KASESE	152	25	24	39	45
Kigali	212	32	14	37	45
Mono	281	21	9	41	45
NAKASONG	31	29	29	26	45
ALAOTRA M	264	24	9	34	45
Kagera	71	37	14	30	45
Central	1840	23	24	32	45
North	1001	30	19	27	45
Rutana	199	37	23	23	45
Grand Gede	141	34	4	35	45
Makamba	234	31	22	28	45
Dar es Salaa	142	36	11	30	44
Pwani	79	34	14	35	44
MUKONO	122	26	19	32	44
LITTORAL	296	33	11	20	44
Simiyu	43	35	9	37	44
ATSINANAN	215	24	7	36	44
Kongo Cent	283	35	14	25	44
Kuntaur	222	36	6	27	44
Marsabit	91	42	4	12	44
KISORO	80	33	20	36	44
Basse	240	38	6	16	44
YAOUNDE	394	37	13	24	44
SIKASSO	512	27	6	37	44
Singida	85	34	15	32	44
Lofa	164	38	5	36	43
Arusha	74	35	22	28	43
Kiambu	88	31	9	27	43
SOFIA	269	23	3	38	43
Bas-Uele	137	36	15	20	43
Baringo	79	28	22	32	43
East	485	34	15	35	43
Western	563	32	17	28	43
BAYELSA	173	31	8	35	43
Luanda	830	33	8	29	43
Murang'a	89	40	7	22	43
Collines	370	18	9	35	42
Mashonalan	665	27	14	31	42
CROSS RIVI	158	24	3	35	42
LUWERO	59	34	19	32	42
OTUKE	45	33	27	31	42
Margibi	147	36	9	29	42
AKWA IBOM	197	32	15	29	42

	Cuanza Nor	419	34	8	24	42
	Zou	434	12	7	38	42
	Muyinga	328	36	17	17	42
	HOIMA	117	27	15	38	42
	Rivercess	117	34	8	33	42
	Iringa	67	31	10	31	42
	Oshikoto	79	24	8	33	42
	KIBAALE	199	32	8	26	42
	Centrale	709	24	9	32	41
	MENABE	262	22	19	31	41
	Morogoro	85	34	15	31	41
	DOUALA	360	30	12	24	41
	Donga	280	20	4	34	41
	SUD-OUEST	117	24	7	35	41
	NORD	557	37	8	22	41
	LWENGO	22	27	9	27	41
	GAO	230	21	8	35	41
	Cunene	300	35	2	14	41
	Atlantic	514	21	8	35	41
	Nyandarua	91	29	9	27	41
	Logone Occ	183	37	7	26	40
	Lindi	77	35	13	29	40
	Otjozondjup	125	26	4	31	40
	GOMBA	20	25	15	25	40
	MASAKA	40	30	15	23	40
	Kilifi	103	28	12	27	40
	Cuanza Sul	423	32	7	24	39
	Mansakonko	193	26	4	31	39
	West Pokot	94	37	7	27	39
	KWARA	207	25	4	36	39
	Bujumbura	209	33	15	21	39
Ethiopia	Oromiya Re	649	31	14	27	39
	IMO	213	28	9	34	39
	Banjul	180	24	8	30	39
	Harari	281	30	4	33	39
	Machakos	93	32	16	23	39
	Masvingo	620	28	10	27	39
	Mwanza	65	29	15	34	38
	ANALANJIR	266	20	8	31	38
	Omaheke	120	28	8	28	38
	Ruyigi	248	31	25	17	38
	SAVA	270	21	9	32	38
	Oueme	412	17	10	33	38
	Nandi	84	27	5	25	38
Malawi	South	2565	24	16	26	38
Mozambiqu	Maputo City	141	30	5	26	38
	Lunda Sul	527	33	8	21	38
	ABIA	181	24	4	29	38
	Siaya	96	33	15	29	38

	Brikama	371	27	7	23	37
	Mandoul	188	31	4	25	37
	Borgou	441	17	11	32	37
	Karas	113	31	5	26	37
	Mwaro	200	28	19	14	37
	KAMPALA	401	24	14	27	37
	KWEEN	19	26	11	21	37
	KALANGAL	87	28	9	26	37
	Alibori	411	27	6	32	37
	Caprivi	131	31	9	25	37
	Moyen Chai	162	30	5	22	36
	Ituri	204	26	7	29	36
	Zaire	491	29	4	27	36
	WAKISO	236	22	15	24	36
	Nyeri	103	23	8	22	36
	Central	703	30	8	18	36
	Kanifing	260	25	3	27	36
	Plateaux	991	16	6	30	36
	Littoral	377	17	7	30	36
	Elgeyo Mara	85	27	9	26	35
	Moxico	250	29	13	21	35
	Khomas	134	20	8	27	35
	Gaza	328	30	4	18	35
	Benishangul	383	22	8	29	35
	Gambella	343	25	8	26	35
	Couffo	332	14	11	29	35
	Bie	403	25	5	22	35
	Kwale	101	28	17	21	35
	N'Djamena	247	23	14	26	34
	Kara	765	24	8	25	34
	BUIKWE	41	27	24	17	34
	North West	518	25	8	21	34
	Lusaka	891	28	7	23	34
	Matabelelan	426	23	6	27	34
	MELAKY	219	13	11	28	34
	EKITI	218	21	5	27	33
	Taita Taveta	78	22	8	24	33
Senegal	Tambacoun	91	23	3	21	33
	Matabelelan	483	18	6	25	33
	Tigray Regi	493	18	12	26	33
	Maputo	193	24	5	22	33
	Bujumbura	168	27	14	20	33
	Amhara Reg	572	21	10	23	33
	VATOVAVY	231	17	4	29	32
	Zambezia	305	21	9	27	32
	Turkana	82	26	9	24	32
	BOENY	231	18	6	26	32
	Hardap	111	23	9	21	32
	Kerewan	242	24	3	21	31

Lome	796	17	8	26	31
SOKOTO	217	6	1	27	30
Isiolo	112	29	4	13	30
DIANA	267	12	5	28	30
BETSIBOKA	263	19	5	26	30
BUYENDE	47	19	15	21	30
Cabinda	391	27	4	11	30
SNNPR	564	18	6	21	29
Dire Dawa	296	20	6	20	29
YOBE	242	8	5	26	29
Erongo	160	19	9	23	29
Maritime	1029	16	6	24	29
Mombasa	91	14	2	20	29
EXTREME-N	634	20	3	19	29
Chari Bagui	173	18	13	18	28
Kajiado	92	20	4	17	28
Manica	295	22	3	18	28
Bomet	94	17	7	20	28
Addis Ababa	288	21	5	20	27
Sedhiou	108	24	1	13	27
Bengo	246	22	4	13	27
TOUMBOU	351	14	4	24	26
Kolda	117	25	2	12	26
Tabora	87	21	9	21	26
LAGOS	318	15	3	21	26
Huambo	471	22	5	17	26
Uige	486	22	4	13	26
Kunene	117	20	4	21	26
Ohangwena	67	21	6	22	25
ADAMAOU	410	18	5	16	25
OSUN	233	18	5	16	25
Fatick	94	17	5	13	24
ATSIMO AN	229	11	3	19	24
Sila	156	17	9	19	24
ZAMFARA	255	9	2	22	24
Kedougou	92	23	5	11	24
Inhambane	224	17	4	14	24
ATSIMO AT	237	11	5	18	24
Sofala	276	18	3	13	24
Batha	136	15	11	13	24
Kaffrine	113	17	3	8	23
Wadi Fira	210	17	9	21	23
Uasin Gishu	102	19	3	14	23
MOPTI	253	16	5	15	23
ONDO	209	17	3	17	22
Lamu	76	18	8	17	22
Borkou	82	15	6	15	22
IHOROMBE	289	15	6	18	22
Kaolack	122	10	6	16	21

Kanem	169	14	14	10	21
Ouaddai	160	14	3	16	21
Afar Region	387	12	3	14	21
ENUGU	183	15	5	19	21
ANDROY	222	10	4	13	21
Cabo Delga	233	15	3	15	21
Dakar	112	13	4	13	21
BORNO	254	11	3	17	20
Guera	211	12	3	15	20
ANOSY	261	7	4	18	20
Wajir	84	15	0	10	20
BUTAMBAL	10	20	0	20	20
Salamat	195	15	4	14	20
Omusati	61	10	7	15	20
FCT ABUJA	231	9	1	16	19
Nampula	359	15	2	12	18
KANO	377	5	3	18	18
Hadjer-Lami	214	10	6	9	18
Ziguinchor	68	9	1	13	18
Mandera	100	16	1	6	17
KEBBI	287	8	0	15	17
Thies	111	14	4	10	16
KATSINA	282	5	3	14	15
OGUN	238	11	1	13	15
Niassa	238	8	5	10	15
Barh El Gaz	158	11	12	11	15
JIGAWA	285	2	2	13	14
Oshana	77	13	1	12	14
NASARAWA	218	9	3	11	14
Ngazidja	1158	6	2	10	14
KIDAL	195	9	3	8	13
OYO	300	8	2	11	12
Cuando Cul	264	10	2	8	12
Diourbel	121	7	5	10	12
Tete	279	10	1	8	11
Moheli	442	5	2	9	11
Lac	212	8	8	3	10
Somali	464	7	0	8	10
Louga	110	6	4	7	10
Garissa	80	8	0	4	9
Saint-Louis	107	5	7	5	8
Ennedi	143	7	4	4	8
Ndzouani	929	5	1	5	8
Matam	102	4	3	4	6
Tibesti	25	4	0	4	4
